# The Forgotten Gate: Choroid Plexus and Blood-CSF Barrier in Arboviral Encephalitis

**DOI:** 10.3390/life16060975

**Published:** 2026-06-09

**Authors:** Cecília M. Wodzik, Matheus Henrique B. Figueiredo, Paula S. Nakamura, Mônica Rodrigues F. Machado, Vivaldo G. da Costa, Rafael M. da Costa, Marielena V. Saivish

**Affiliations:** 1 Laboratory of Fish Physiology and Biotechnology (LabFISH), Department of Biosciences, Federal University of Jataí (UFJ), Jataí 75801-615, GO, Brazil; cecilia.mullerwodzik@gmail.com (C.M.W.); matheus.barcelosf@gmail.com (M.H.B.F.); paulanakamura82@gmail.com (P.S.N.); monica_rodrigues@ufj.edu.br (M.R.F.M.); 2 Genomics Laboratory, Department of Biology, São Paulo State University (UNESP), São José do Rio Preto 15054-000, SP, Brazil; vivaldo14@gmail.com; 3 Laboratory of Cardiovascular Pharmacology and Toxicology (LAFTOCAR), Department of Health Sciences, Federal University of Jataí (UFJ), Jataí 75801-615, GO, Brazil; rafael_menezes@ufj.edu.br; 4 Laboratory of Virology Research, Department of Dermatological, Infectious and Parasitic Diseases, School of Medicine of São José do Rio Preto (FAMERP), São José do Rio Preto 15090-000, SP, Brazil

**Keywords:** arboviral encephalitis, choroid plexus, blood-cerebrospinal fluid barrier, BCSFB, blood-brain barrier, neuroinvasion, neuroinflammation, cerebrospinal fluid, flavivirus, central nervous system

## Abstract

Mechanisms of arboviral neuroinvasion are still incompletely resolved, despite longstanding emphasis on the blood-brain barrier (BBB) as the principal interface for central nervous system (CNS) entry. While BBB-centered models have been highly informative, they may underrepresent the contribution of other CNS border structures, particularly the choroid plexus and the blood-cerebrospinal fluid barrier (BCSFB). Here, we re-examine the BCSFB as a relevant but unevenly supported neuroinvasion interface in arboviral encephalitis. The strongest direct evidence is currently available for Zika virus (ZIKV), for which experimental studies support infection of choroid plexus-associated cells and CNS access through the blood-CSF axis. Semliki Forest virus (SFV) provides additional direct, although still limited, support for this concept. In contrast, for West Nile virus (WNV), Japanese encephalitis virus (JEV), and tick-borne encephalitis virus (TBEV), evidence for choroid plexus involvement remains indirect or insufficiently resolved, even though neuroinvasion itself is well established. We therefore argue not for replacement of BBB-centered models, but for broader integration of the BCSFB into current frameworks of arboviral CNS invasion. This evidence-based perspective supports a hierarchical, virus-dependent view of choroid plexus involvement and highlights the need for mechanistic studies that directly test when and how this interface contributes to encephalitic disease.

## 1. Introduction

Neuroinvasive arboviral disease remains one of the most severe manifestations of infection by mosquito- and tick-borne RNA viruses, yet the anatomical routes by which these pathogens access the central nervous system (CNS) have not been completely established [1,2,3,4,5]. Much of the field has been shaped by a predominantly blood-brain barrier (BBB)-centered framework, in which neuroinvasion is discussed in terms of endothelial infection, inflammatory barrier disruption, paracellular leakage, leukocyte-assisted trafficking, or retrograde neuronal spread [1,2,3,4,5]. That framework has been highly valuable, but it does not encompass the full range of CNS border structures that may participate in viral entry [1,6]. In particular, it has left comparatively little room for the choroid plexus and the blood-cerebrospinal fluid barrier (BCSFB), despite their strategic anatomical position and immunological activity at the interface between blood, cerebrospinal fluid (CSF), and the brain [6,7,8,9].

The choroid plexus is not merely a CSF-producing tissue [6,8]. It is a specialized neuroepithelial structure composed of fenestrated stromal capillaries, resident immune and stromal elements, and a polarized epithelial layer sealed by tight junctions that forms the BCSFB [6,8,9]. Because this barrier differs from the non-fenestrated endothelial architecture of the BBB, it is likely to impose distinct constraints on pathogen exposure, cellular tropism, immune sensing, and barrier crossing [6,8]. In parallel, the choroid plexus is increasingly recognized as an active participant in CNS immune surveillance, regulating cytokine signaling and leukocyte trafficking under both homeostatic and inflammatory conditions [7,9]. Taken together, these properties support the BCSFB as a potential site of arbovirus-host interaction, even if its contribution is unlikely to be uniform across all neurotropic viruses [6,7,8,9].

Nonetheless, the current evidentiary landscape remains uneven, and that asymmetry should be stated explicitly. Among arboviruses, the strongest direct support for BCSFB involvement has emerged in Zika virus (ZIKV) infection [10]. Experimental work has shown that ZIKV can infect pericytes in the choroid plexus and reach the CNS through the blood-CSF interface, with data further suggesting that virus present in the CSF may contribute to subsequent brain infection [10]. More recently, Semliki Forest virus (SFV) has provided additional direct support for the concept, with evidence that neuroinvasion can occur through the BCSFB in a manner dependent on VLDLR-expressing choroid plexus epithelial cells [11]. These studies do not support a shared arboviral mechanism, but they do demonstrate that the choroid plexus is more than a theoretical bystander in selected viral systems [10,11].

For other clinically important arboviruses, the evidence is less well defined. In West Nile virus (WNV), Japanese encephalitis virus (JEV), and tick-borne encephalitis virus (TBEV), neuroinvasion is well established, but the specific contribution of the choroid plexus and the BCSFB remains incompletely defined [1,2,3,4,5]. In these infections, the literature has focused primarily on BBB-associated mechanisms, inflammatory amplification, cellular trafficking, and neuronal routes, while evidence for a primary BCSFB-mediated pathway is still limited, indirect, or insufficiently tested [1,2,3,4,5]. The current literature does not justify treating the choroid plexus as a universally established gateway for arboviral CNS entry; rather, it supports a more cautious view in which the relevance of the BCSFB is likely virus-dependent and still underexplored outside a small number of experimental models [1,2,3,4,5].

This review therefore does not seek to replace BBB-centered models with a new single-route paradigm. Instead, it re-examines the choroid plexus and the BCSFB as underappreciated CNS border interfaces whose relevance should be interpreted according to the available experimental support for each virus [1,6,10,11]. By distinguishing directly supported findings from indirect or conceptual extrapolation, we aim to place the BCSFB more accurately within current models of arboviral encephalitis and to clarify where current evidence is compelling, where it remains preliminary, and where critical experimental gaps still persist [1,6,10,11].

## 2. Materials and Methods

### Search Strategy and Evidence Synthesis

This article was developed as a structured narrative review focused on the potential role of the choroid plexus and the blood-cerebrospinal fluid barrier (BCSFB) in arboviral neuroinvasion. A targeted literature search was conducted in PubMed, Scopus, and Web of Science to identify studies relevant to arboviral encephalitis, neuroinvasion pathways, choroid plexus biology, BCSFB function, cerebrospinal fluid biomarkers, and experimental models of CNS barriers. Search terms included combinations of “arbovirus,” “arboviral encephalitis,” “neuroinvasion,” “choroid plexus,” “blood-cerebrospinal fluid barrier,” “blood-CSF barrier,” “blood-brain barrier,” “cerebrospinal fluid,” “Zika virus,” “West Nile virus,” “Japanese encephalitis virus,” “tick-borne encephalitis virus,” “Semliki Forest virus,” “flavivirus,” “alphavirus,” “neuroinflammation,” “barrier dysfunction,” “single-cell transcriptomics,” and “choroid plexus immune response.”

Priority was given to primary experimental studies that directly investigated viral interactions with the choroid plexus, BCSFB integrity, CSF-associated viral dissemination, barrier-level host-pathogen dynamics, or mechanisms of CNS entry. Foundational reviews and broader conceptual papers were included when necessary to provide anatomical, immunological, diagnostic, or methodological context [1,6,8,9,12,13]. Additional references were identified through citation tracking of key articles. Because this was not designed as a systematic review, no formal meta-analysis, risk-of-bias assessment, or exhaustive screening protocol was performed. However, studies were interpreted according to the strength of evidence supporting choroid plexus or BCSFB involvement. For the purposes of this review, “direct evidence” was defined as experimental demonstration of viral infection, traversal, receptor usage, or functional barrier disruption involving choroid plexus or BCSFB components. “Indirect evidence” was defined as evidence compatible with choroid plexus or BCSFB involvement, such as CSF abnormalities, periventricular inflammation, leukocyte recruitment, or barrier permeability changes, without direct demonstration that this interface served as a route of viral entry. “Speculative or hypothetical evidence” was defined as biological plausibility based on receptor expression, barrier physiology, or non-arboviral models, without direct validation in arboviral infection. The literature was synthesized thematically, with emphasis on distinguishing experimentally supported mechanisms from indirect inference and conceptual extrapolation. This approach was used to avoid treating the choroid plexus as a universal gateway for arboviral neuroinvasion and to instead evaluate its potential role within a virus-dependent and evidence-based framework [1,2,10,11].

## 3. Main Concepts and Evidence

### 3.1. Anatomy and Biology of the Choroid Plexus and the Blood-CSF Barrier

**Figure 1:**
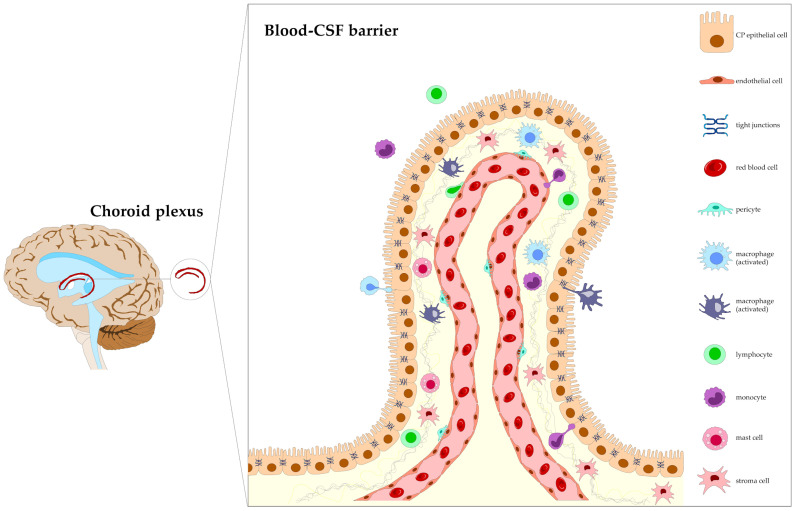
Schematic representation of the epithelial-vascular organization of the choroid plexus and the blood-cerebrospinal fluid barrier (BCSFB). Fenestrated stromal capillaries allow the passage of solutes and circulating molecules into the stromal compartment, while epithelial cells interconnected by tight junctions establish the principal restrictive barrier regulating exchange with the cerebrospinal fluid (CSF). The stromal region contains resident and infiltrating immune cells, including macrophages, dendritic cells, lymphocytes, and monocytes, supporting the role of the choroid plexus as an immunologically active interface between the peripheral circulation and the central nervous system. Structural and functional differences between the BCSFB and the blood-brain barrier (BBB) may influence pathogen exposure, immune signaling, and susceptibility to neuroinvasion.

The choroid plexus forms the structural and functional basis of the BCSFB and represents a key interface regulating molecular exchange and immune interactions between the systemic circulation and the CNS. Its primary physiological functions include the production of CSF and the regulation of molecular transport between the systemic circulation and the ventricular compartment (Figure 1).

The stroma of the choroid plexus contains capillaries with fenestrations of approximately 60 to 80 nm, conferring high permeability to the vascular component [6]. This compartment also includes connective tissue and immune cells, such as antigen-presenting cells, which participate in local immune surveillance. However, the barrier function is not performed by the capillary endothelium but by the choroid epithelium, whose cells are connected by tight junction complexes that restrict paracellular diffusion and establish selective permeability over the cerebrospinal fluid microenvironment [12]. The average volume of CSF in healthy individuals is approximately 150 mL. This fluid plays central roles in maintaining the CNS microenvironment, including regulation of ionic balance, removal of metabolites, and distribution of signaling molecules. Its composition reflects both physiological and pathological processes, supporting its use as a clinical sample in diagnostic investigations [14]. In addition, alterations in its composition may provide indirect evidence of dysfunction in central nervous system barriers, including the BCSFB.

In contrast, the BBB is formed by non-fenestrated endothelial cells of cerebral capillaries, associated with tight junction complexes, pericytes, and astrocytic end-feet, collectively constituting the neurovascular unit. This structural organization establishes a selective system for the transport of substances between the blood and the brain parenchyma. The BBB plays a central role in maintaining neuronal homeostasis by restricting the entry of potentially harmful compounds and regulating the supply of essential nutrients [15]. However, this high degree of structural restriction contrasts with the organization of the BCSFB, in which selectivity is predominantly determined by the choroid epithelium, rich in fenestrated blood vessels, suggesting important functional differences in interactions with circulating pathogens. These structural and functional differences highlight that multiple CNS interfaces may interact with circulating pathogens; however, the interpretation of neuroinvasion has historically been dominated by a BBB-centered framework.

### 3.2. Why the Field Became BBB-Centric

The predominance of the BBB in models of viral neuroinvasion reflects historical and methodological factors that have shaped the development and interpretation of the field. Since its early conceptualization as a highly selective protective interface separating the CNS from systemic circulation, the BBB has been regarded as the primary gateway controlling molecular and cellular exchange with the brain. The consolidation of the neurovascular unit concept further reinforced its central role as the dominant framework for studying CNS homeostasis and pathology [16,17,18]. This conceptual centrality has been reinforced by experimental approaches. A large proportion of studies investigating neuroinvasion rely on in vitro models based on brain microvascular endothelial cells or in vivo systems focused on vascular permeability, leukocyte trafficking, and tight junction integrity. These approaches have established endothelial dysfunction and increased BBB permeability as primary readouts of CNS invasion. In contrast, the choroid plexus and the BCSFB have been underrepresented, partly due to their structural complexity, limited accessibility, and the relative scarcity of standardized experimental models capable of reproducing their epithelial-stromal organization and immune environment [17,19].

Although the BBB and the BCSFB are frequently grouped as neuroimmune barriers, their differences in anatomical and physiological organization imply distinct consequences for neuroinvasion. Whereas the BBB is composed of continuous microvascular endothelial cells interconnected by highly restrictive tight junctions, the BCSFB is characterized by vascularized stromal structures and a fenestrated epithelium containing resident immune cells and pronounced secretory activity. This distinction suggests that the choroid plexus is more closely associated with immunological functions linked to systemic inflammatory signaling, differing from the traditionally dominant experimental focus centered on the BBB [16,19].

Within this BBB-centered framework, a set of canonical mechanisms of neuroinvasion has been established and widely adopted. These include transcellular transport across endothelial cells, paracellular leakage associated with inflammation-induced disruption of tight junctions, immune cell-mediated transport (“Trojan horse” mechanism), and cytokine and matrix metalloproteinase-mediated barrier dysfunction. Together, these mechanisms have provided the dominant framework for interpreting viral entry into the CNS for decades [20]. Emerging evidence indicates that this model does not fully account for all observed patterns of neuroinvasion. In several viral infections, including arboviral models, viral presence in the CNS has been detected in the absence of overt BBB disruption. Additional findings, such as early detection of viral components in the CSF and preferential involvement of periventricular regions, suggest that alternative interfaces may contribute to viral entry and dissemination [21].

These observations suggest that the historical emphasis on the BBB has left other CNS interfaces largely underexplored, including the choroid plexus and the BCSFB. Reassessing these structures is therefore justified, not as a replacement for BBB-centered models, but as a way to test whether current frameworks have incompletely captured the range of neuroinvasion routes used by different neurotropic arboviruses.

### 3.3. Evidence for Choroid Plexus Involvement in Arboviral Infections

Evidence supporting involvement of the choroid plexus and the BCSFB in arboviral neuroinvasion remains uneven and should not be interpreted as equivalent across viral systems (Table 1). The strongest direct experimental support is currently available for ZIKV, for which experimental work has shown infection of choroid plexus-associated pericytes and CNS access through the blood-CSF interface [10]. SFV provides a more recent and mechanistically important example, with evidence that neuroinvasion can occur through the BCSFB via VLDLR-expressing choroid plexus epithelial cells [11]. In contrast, for WNV, JEV, and TBEV, the available literature primarily supports established neuroinvasion through BBB-associated disruption, inflammatory amplification, leukocyte trafficking, or neuronal routes, whereas the specific contribution of the choroid plexus remains indirect, incompletely resolved, or speculative [1,2,3,4,5]. Therefore, the current literature supports a hierarchical interpretation rather than a universal model in which all arboviruses use the BCSFB through similar mechanisms [1,2,10,11].

Among the arboviral infections discussed in this review, ZIKV currently provides the clearest direct evidence for choroid plexus and BCSFB involvement. Experimental data indicate that ZIKV can infect pericytes in the choroid plexus and that early virus in the CSF may contribute to subsequent brain infection [10]. These findings support the interpretation that the blood-CSF axis may participate directly in ZIKV neuroinvasion, although the precise sequence of stromal infection, epithelial barrier dysfunction, CSF release, and later brain parenchymal infection remains incompletely resolved [10]. SFV provides additional direct evidence that the BCSFB can function as a neuroinvasion route in at least one alphaviral system. Recent work showed that intravenously administered SFV required VLDLR for neuroinvasion and that the virus primarily entered the CNS through the BCSFB by infecting VLDLR-expressing choroid plexus epithelial cells [11]. This finding strengthens the broader concept that the choroid plexus can serve as a functional route of viral CNS entry. However, this evidence should not be generalized to all arboviruses without virus-specific validation [11].

The situation is considerably less resolved for WNV, JEV, and TBEV. WNV neuroinvasion has been extensively studied, but dominant models emphasize BBB dysfunction, hematogenous spread, inflammatory permeability changes, leukocyte-associated trafficking, and neuronal routes, while the role of the blood-CSF barrier remains comparatively underexplored [2]. JEV neuroinvasion has similarly been discussed in relation to inflammatory responses, interactions with host cells, immune-cell-associated spread, and mechanisms of entry into the brain, but direct BCSFB traversal through the choroid plexus has not been clearly established [3]. For TBEV, experimental evidence supports BBB permeability changes during infection, but this does not establish the BCSFB as a primary entry route [4,5]. Taken together, the available evidence supports a cautious and virus-dependent model of choroid plexus involvement in arboviral encephalitis. In ZIKV infection, direct experimental evidence places the choroid plexus and BCSFB within the neuroinvasion process [10]. In SFV infection, receptor-based evidence involving VLDLR-expressing choroid plexus epithelial cells provides additional direct support, although this remains limited to specific experimental models [11]. For WNV, JEV, and TBEV, the choroid plexus should not yet be presented as a confirmed primary gateway. In these infections, BCSFB involvement remains biologically plausible but experimentally unresolved [1,2,3,4,5]. This distinction is important because CSF abnormalities, periventricular inflammation, or barrier dysfunction may reflect secondary inflammatory effects rather than direct viral entry through the choroid plexus [6,12,13].

**Table 1:** Current evidence supporting the involvement of the choroid plexus and blood-cerebrospinal fluid barrier (BCSFB) in arboviral neuroinvasion. Evidence is classified as direct, indirect, or speculative according to whether the cited studies experimentally demonstrate choroid plexus/BCSFB involvement, only suggest it through associated findings, or propose biologically plausible but unvalidated mechanisms.

Virus	Evidence Level for Choroid Plexus/BCSFB Involvement	Main Evidence Relevant to the Choroid Plexus or BCSFB	Experimental Models	Cell Types or Structures Implicated	Main Limitations
*ZIKV*	Direct evidence	ZIKV has been shown experimentally to infect choroid plexus-associated pericytes and to access the CNS through the blood-CSF interface. Early viral presence in the CSF may contribute to subsequent brain infection [10].	In vivo murine models and in vitro BCSFB-related experimental systems [10].	Choroid plexus pericytes and choroid plexus barrier-associated cells [10].	The precise sequence connecting stromal infection, epithelial dysfunction, viral release into the CSF, and later parenchymal infection remains incompletely resolved [10].
*SFV*	Direct but still limited evidence	SFV neuroinvasion has been shown to depend on VLDLR and to occur primarily through the BCSFB via infection of VLDLR-expressing choroid plexus epithelial cells [11].	In vivo murine models, receptor-focused approaches, and genetic knockout strategies [11].	VLDLR-expressing choroid plexus epithelial cells [11].	Evidence is strong within the specific experimental system, but relevance to other arboviruses and human infection requires further validation [11].
*WNV*	Indirect or unresolved evidence	WNV neuroinvasion is well established, but most evidence emphasizes BBB dysfunction, inflammatory permeability changes, leukocyte-associated trafficking, and neuronal routes. The blood-CSF barrier remains comparatively underexplored [2].	In vivo models, BBB-focused studies, and neuroinvasion/pathogenesis studies [2].	Endothelial cells, leukocytes, neurons, and possibly other CNS interface-associated cells [2].	Direct choroid plexus traversal has not been sufficiently demonstrated; BBB and BCSFB contributions are difficult to separate [2].
*JEV*	Indirect or unresolved evidence	JEV entry into the brain has been discussed in relation to inflammatory responses, host-cell interactions, immune-cell-associated spread, and intercellular dissemination, but direct BCSFB traversal remains insufficiently established [3].	In vivo and in vitro infection/pathogenesis models [3].	Endothelial cells, neurons, microglia, immune cells, and potentially barrier-associated compartments [3].	Direct infection or traversal of choroid plexus epithelial cells has not been clearly demonstrated [3].
*TBEV*	Indirect/speculative evidence	TBEV infection can increase BBB permeability in mice, but this evidence does not establish the choroid plexus or BCSFB as a primary entry route [4,5].	In vivo murine models and broader pathogenesis studies [4,5].	BBB-associated structures, inflammatory mediators, neurons, and possibly other CNS interface-associated compartments [4,5].	Evidence for BCSFB-mediated entry remains unresolved; BBB dysfunction should not be interpreted as proof of choroid plexus traversal [4,5].

### 3.4. Mechanisms of Viral Crossing of the Blood-CSF Barrier

**Figure 2:**
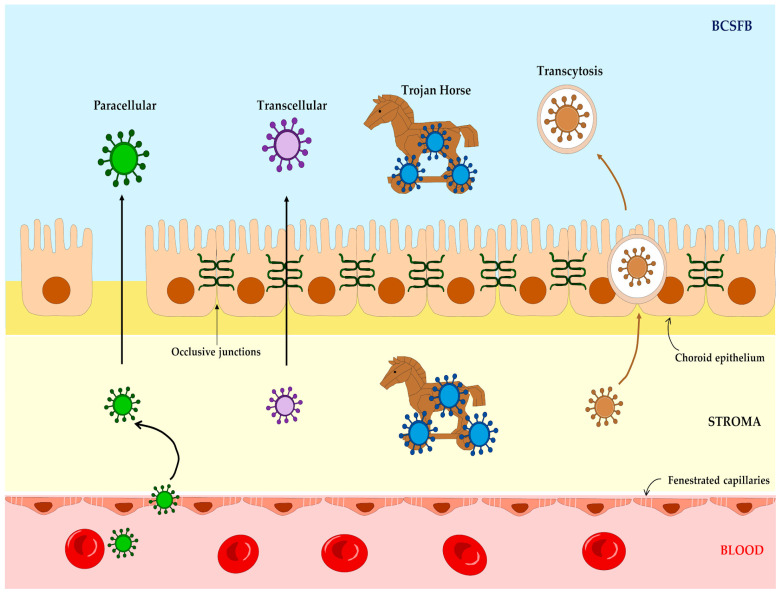
Schematic representation of proposed pathways by which neurotropic viruses may traverse the BCSFB. Potential mechanisms include paracellular transport associated with modulation or disruption of epithelial tight junctions, transcellular passage through infected epithelial cells, vesicle-mediated transcytosis, and immune cell-mediated trafficking (“Trojan horse” mechanism). Additional routes may involve infection of stromal components, including pericytes and resident immune cells, preceding viral access to the CSF. The relative contribution of each pathway likely varies according to viral tropism, inflammatory context, host immune status, and stage of infection. Although some mechanisms are supported experimentally in selected viral systems, others remain hypothetical or incompletely validated in arboviral infections.

Several mechanisms may theoretically allow neurotropic viruses to interact with or traverse the BCSFB, including transcellular infection, paracellular passage after tight junction disruption, vesicle-mediated transcytosis, immune cell-mediated trafficking, and infection of stromal components of the choroid plexus [6,12,13]. However, these mechanisms are not equally supported in arboviral infections. Some have been directly demonstrated only in selected viral systems, whereas others remain inferred from general CNS barrier biology, non-arboviral infection models, or broader inflammatory studies [6,12,13,22,23]. Therefore, the mechanisms discussed below should be interpreted according to their level of experimental support rather than as established pathways shared by all arboviruses. The diversity of these mechanisms suggests that BCSFB crossing occurs through complementary and dynamic routes, influenced by viral type and host inflammatory status, as illustrated in Figure 2.

Transcellular traversal refers to direct infection of barrier-forming cells followed by viral release toward the CSF compartment. In the context of arboviral neuroinvasion, direct evidence for this route remains limited. In ZIKV infection, experimental evidence supports infection of choroid plexus-associated pericytes and suggests viral access to the CNS through the blood-CSF interface [10]. In SFV infection, recent evidence indicates that VLDLR-expressing choroid plexus epithelial cells can mediate neuroinvasion through the BCSFB [11]. For most other arboviruses, direct epithelial traversal through the choroid plexus has not been sufficiently demonstrated [1,2,3,4,5]. Paracellular passage may occur when inflammatory mediators alter tight junction organization and increase epithelial permeability. The BCSFB is formed by choroid plexus epithelial cells connected by tight junction complexes, and inflammatory or infectious stimuli can compromise barrier integrity in experimental models [6,12,13,22,23]. However, in arboviral encephalitis, it remains difficult to determine whether paracellular leakage represents a primary route of viral entry or a secondary consequence of systemic and CNS inflammation [1,2,6,12]. This distinction is important because barrier disruption observed during infection does not necessarily prove that the BCSFB served as the initial anatomical route of neuroinvasion [1,2,6].

Immune cell-mediated transport, often described as a “Trojan horse” mechanism, involves the migration of infected leukocytes across CNS interfaces. The choroid plexus and BCSFB can participate in immune-cell recruitment, leukocyte trafficking, and compartmentalized immune responses within the CNS [9,12,13,24]. In arboviral disease, leukocyte-associated trafficking is frequently discussed as a possible neuroinvasion mechanism, particularly in relation to WNV and JEV pathogenesis [2,3]. However, direct evidence that this mechanism specifically mediates arboviral traversal through the BCSFB remains limited [2,3,12,13]. Infection of stromal components, particularly pericytes and resident immune cells, represents another possible mechanism. This pathway is most directly supported by ZIKV, for which infection of choroid plexus pericytes has been experimentally reported [10]. Stromal infection could create a local viral reservoir and promote inflammatory or structural changes that facilitate viral access to the CSF [10]. Nevertheless, whether stromal infection alone is sufficient for productive BCSFB crossing, and whether this mechanism applies broadly to other arboviruses, remains unclear [1,6,10,12]. Receptor-mediated transcytosis is biologically plausible but currently speculative in the context of arboviral BCSFB traversal. Receptors expressed in the choroid plexus, including FcRn and LRP2, participate in transport processes and may theoretically influence movement of therapeutic molecules, proteins, immune complexes, or potentially viral particles across epithelial barriers [23,25]. However, direct experimental evidence demonstrating FcRn- or LRP2-mediated arboviral neuroinvasion through the BCSFB is currently lacking. These pathways should therefore be presented as hypothetical and not as validated mechanisms in arboviral infections [23,25].

The local immune response of the choroid plexus may influence several of these potential routes. Choroid plexus epithelial and stromal cells can participate in immune surveillance, cytokine signaling, chemokine production, and leukocyte recruitment [9,12,13,24]. This immune activation may restrict viral replication but may also increase barrier permeability and promote leukocyte trafficking [9,12,13,24]. Thus, the choroid plexus should be understood as a dynamic immune-barrier interface rather than as a passive anatomical gate [6,9,12,13]. The interpretation of these mechanisms depends strongly on the experimental model used. Two-dimensional epithelial monolayers allow controlled analysis of permeability and tight junction integrity but lack vascular, stromal, and immune complexity [23,26,27]. Choroid plexus organoids can reproduce aspects of epithelial polarity, selective barrier function, and CSF-like fluid production, but they often lack mature vascularization and immune components [28]. Animal models permit integrated analysis of systemic inflammation, viral dissemination, and neuroinvasion, but interspecies differences limit direct extrapolation to human disease [1,6,29]. These limitations help explain why the relative contribution of each BCSFB crossing mechanism remains unresolved.

Future studies should combine temporal viral tracking, CSF analysis, barrier permeability assays, cell-type-specific infection mapping, receptor validation, and single-cell or spatial transcriptomic approaches. Such studies are needed to determine whether BCSFB involvement is primary, secondary, contributory, or merely associated with broader inflammatory changes during arboviral encephalitis [6,10,11,12,13,24,27].

### 3.5. The Choroid Plexus as an Immune Signaling Hub

The choroid plexus is increasingly recognized as an active neuroimmune interface rather than only a CSF-producing structure. Its epithelial, stromal, vascular, and immune compartments contribute to immune surveillance, cytokine sensing, leukocyte trafficking, and regulation of the CSF inflammatory environment [6,8,9,12,13,24]. This is relevant to arboviral encephalitis because the choroid plexus is anatomically positioned to detect systemic inflammatory signals and to shape immune communication between blood, CSF, and brain-associated compartments [6,9,12,13]. Recent studies have shown that the choroid plexus contains heterogeneous epithelial, stromal, endothelial, macrophage, dendritic-cell, and lymphocyte-associated populations [12,13,24]. Choroid plexus immune cells, especially macrophage populations, contribute to barrier function, CSF cytokine regulation, and neuroimmune responses [24]. In addition, the BCSFB has been described as an interface capable of recruiting, modulating, and suppressing immune cells within the CNS environment [9]. These findings support the concept that the choroid plexus can participate in compartmentalized immune responses, although much of this evidence derives from broader neuroinflammatory, infectious, aging, autoimmune, or non-arboviral contexts rather than from direct arboviral models [9,12,13,24,30].

During viral infection or systemic inflammation, activation of type I and type II interferon pathways may induce JAK/STAT signaling, phosphorylation of STAT1/STAT2, and expression of interferon-stimulated genes [9,12,13,24]. These responses may restrict viral replication and contribute to antiviral defense. At the same time, inflammatory activation can induce chemokines such as CCL2 and CXCL10 and cytokines such as TNF-α, IL-1β, and IL-6, which may promote leukocyte recruitment and alter barrier permeability [9,12,13,24]. Therefore, immune signaling at the choroid plexus may have a dual effect: it may protect the CNS by limiting viral spread, but it may also contribute to inflammatory barrier dysfunction when excessive or poorly regulated [9,12,13,24]. In arboviral encephalitis, this immune-hub model remains plausible but incompletely validated. It is currently better supported as a framework for generating testable hypotheses than as a fully established mechanism of disease [1,2,6,12,13]. For ZIKV and SFV, direct evidence supports involvement of the choroid plexus/BCSFB axis in neuroinvasion [10,11]. For WNV, JEV, TBEV, and other arboviruses, the role of choroid plexus immune signaling remains less clearly defined and should be interpreted as a potential contributor rather than a proven driver of neuroinvasion [1,2,3,4,5].

**Figure 3:**
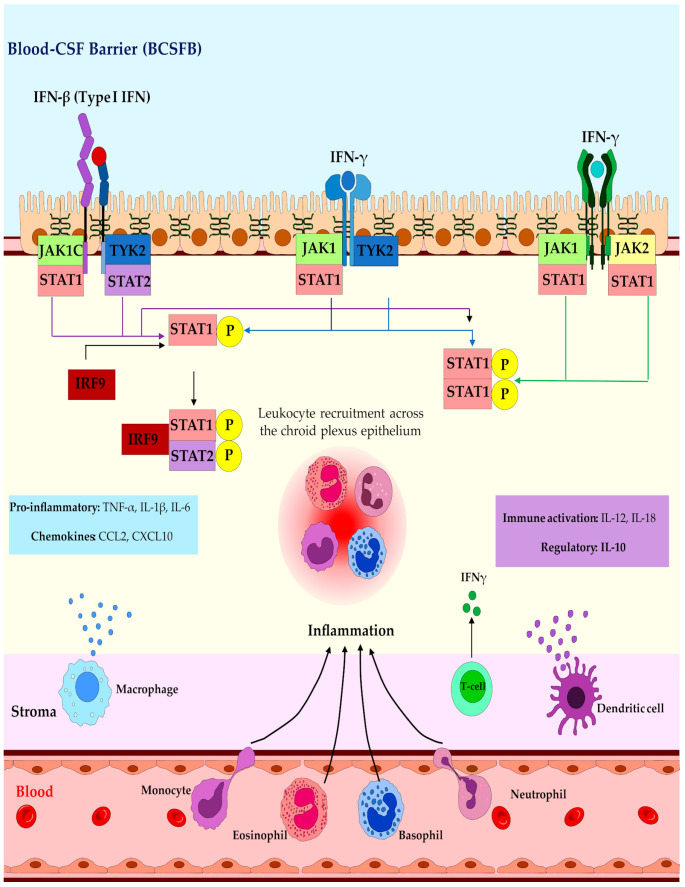
Choroid plexus immune signaling and proposed neuroinvasion-associated processes during arboviral infection. Viral exposure or systemic inflammatory stimulation may activate type I and type II interferon pathways in choroid plexus epithelial, stromal, and immune-cell populations, leading to JAK/STAT signaling, induction of interferon-stimulated genes, cytokine production, and chemokine-mediated leukocyte recruitment [9,12,13,24]. These responses may contribute to antiviral defense but may also alter BCSFB permeability and facilitate inflammatory cell migration into the CSF [9,12,13,24]. The figure illustrates several possible routes of viral interaction with the choroid plexus, including stromal infection, epithelial traversal, immune cell-mediated trafficking, and cytokine-associated barrier modulation. Several mechanisms shown remain partially characterized or hypothetical in arboviral encephalitis and should not be interpreted as equally validated for all viruses [1,2,6,10,11,12,13].

Thus, the choroid plexus should not be regarded solely as a barrier, but as a dynamic component that integrates systemic inflammatory signals, local immune responses, and neuroinvasion processes (Figure 3).

### 3.6. Diagnostic Implications

Diagnostic evaluation of arboviral CNS infection is clinically important for confirming neuroinvasive disease and characterizing intrathecal inflammation. However, current diagnostic tools have limited ability to identify the anatomical route by which a virus entered the CNS [31,32]. This limitation is central to the interpretation of the choroid plexus and BCSFB in arboviral encephalitis. Common diagnostic approaches include detection of viral RNA in CSF by RT-PCR, detection of intrathecal IgM or other virus-specific antibodies, and assessment of inflammatory changes such as lymphocytic pleocytosis, moderate protein elevation, and increased concentrations of cytokines or chemokines [31,32]. These findings can support the diagnosis of neuroinvasive infection, but they do not distinguish whether viral entry or inflammatory amplification occurred primarily through the BBB, the BCSFB, or another CNS interface [6,12,31,32].

CSF abnormalities may be compatible with choroid plexus or BCSFB involvement, especially when viral RNA, inflammatory mediators, or immune cells are detected early in the disease course [10,12,13,31]. However, such findings are not specific. Viral RNA in CSF may reflect direct access through the BCSFB, secondary spread from infected brain tissue, BBB disruption, leukocyte-associated trafficking, or inflammatory propagation between CNS compartments [1,2,6,10,12]. Similarly, intrathecal cytokines and chemokines may reflect local choroid plexus activation, but they may also arise from meningeal, perivascular, glial, or parenchymal immune responses [9,12,13,24]. At present, no validated clinical biomarker can reliably distinguish primary BCSFB dysfunction from BBB disruption or secondary systemic inflammation in arboviral encephalitis [6,12,31,32]. This is an important translational gap. A more informative diagnostic approach would require paired serum and CSF analysis, temporal sampling, viral load kinetics, barrier-specific injury markers, neuroimaging, CSF immune profiling, and experimental correlation with tissue-level changes in the choroid plexus and BBB [6,12,13,24,31,32]. Therefore, the diagnostic relevance of the BCSFB should be framed cautiously. Current CSF tests are useful for confirming neuroinvasive arboviral disease, but they cannot define the precise route of neuroinvasion [31,32]. Future studies should aim to identify biomarker combinations that can distinguish BBB-associated injury from BCSFB-associated dysfunction and determine whether specific CSF signatures correlate with choroid plexus infection, epithelial injury, or compartmentalized immune activation [6,12,13,24].

### 3.7. Therapeutic Implications

The choroid plexus and BCSFB may represent future therapeutic targets for limiting viral neuroinvasion, modulating neuroinflammation, or improving CNS drug delivery. However, in the context of arboviral encephalitis, these applications remain largely conceptual. No validated BCSFB-specific antiviral or barrier-protective therapy is currently established for arboviral CNS disease [1,2,6,12,25]. One potential strategy is modulation of inflammation-induced barrier dysfunction. Pro-inflammatory cytokines and chemokines can alter barrier integrity, promote leukocyte recruitment, and increase permeability at CNS interfaces [6,9,12,13,24]. In theory, carefully timed immunomodulation could help preserve BCSFB integrity or limit damaging neuroinflammation. However, this possibility requires caution. In viral encephalitis, inflammatory responses may contribute both to viral control and to tissue injury [1,2,12,13]. Therefore, broad immunosuppression, including corticosteroid-based approaches, may have different effects depending on virus, disease stage, host immune status, and treatment timing. The therapeutic value of targeting BCSFB inflammation in arboviral encephalitis remains unproven and requires direct experimental validation [1,2,6,12].

A second potential application involves drug delivery through the choroid plexus. Because the BCSFB regulates molecular exchange between blood and CSF, receptor-mediated transport pathways at this interface could theoretically be used to deliver antiviral or neuroprotective compounds into the CSF [23,25]. Receptors such as FcRn and LRP2 have been discussed in relation to transport biology and protein drug delivery, but their role in arboviral neuroinvasion or arbovirus-specific therapeutic delivery has not been demonstrated [23,25]. These pathways should therefore be considered speculative in the present context [23,25]. Intrathecal or intraventricular administration can bypass some physiological barriers and increase drug exposure within the CSF [33]. However, these approaches are invasive and may be limited by infection risk, uneven drug distribution, CSF clearance, and uncertain penetration into brain parenchyma [33]. In addition, CSF delivery does not automatically ensure effective concentrations at infected neural or glial targets [25,33]. Overall, the therapeutic implications of the BCSFB in arboviral encephalitis should be presented as an emerging research direction rather than an established intervention strategy. Future studies should determine whether preserving BCSFB integrity, blocking virus-specific entry pathways, modulating local immune signaling, or optimizing CSF-directed drug delivery can meaningfully reduce CNS infection or neurological injury [1,2,6,10,11,12,25].

### 3.8. Experimental Models to Study the Blood-CSF Barrier

In vitro models based on choroid plexus epithelial cells enable the analysis of BCSFB transport and permeability under controlled conditions [23,26,27]. In Transwell systems, primary cells or immortalized cell lines are cultured on permeable membranes, forming compartments that simulate the blood-facing and CSF-facing domains [23,26]. Barrier integrity can be assessed through transepithelial electrical resistance and molecular permeability assays [23,26]. These systems are reproducible and useful for functional quantification, but they do not fully capture the multicellular, vascular, stromal, immune, and flow-related complexity present in vivo [23,27]. Three-dimensional models derived from pluripotent stem cells partially reproduce the structural organization of the choroid plexus [28]. Choroid plexus organoids can exhibit polarized epithelium, selective barrier properties, and internal compartments containing CSF-like fluid [28]. However, the absence of mature vascularization and immune components limits their ability to reproduce the full BCSFB microenvironment [27,28].

Microfluidic and organ-on-chip approaches may increase physiological relevance by incorporating flow, compartmentalization, and multicellular interactions [23,27]. However, BCSFB-specific applications remain less standardized than BBB-focused platforms, and these systems are limited by technical complexity, cost, and differences among protocols [23,27]. Therefore, BBB-on-chip studies should not be used as direct substitutes for BCSFB models unless the distinction between the two barriers is explicitly stated [23,27,34]. Animal models enable integrated evaluation of viral dissemination, systemic inflammation, immune responses, barrier dysfunction, and neuroinvasion within a living organism [1,6,10,11,29]. However, interspecies differences in choroid plexus biology, receptor expression, immune responses, and viral tropism limit direct extrapolation to human disease [1,6,29]. Collectively, each model presents specific limitations. Two-dimensional systems are highly reproducible but simplified [23,26]. Organoids provide three-dimensional epithelial organization and CSF-like fluid production but lack mature vascular and immune compartments [28]. Microfluidic platforms increase physiological fidelity but require specialized infrastructure and standardization [23,27]. Animal models enable systemic analysis but are constrained by species-specific differences [1,6,29]. Integration of multiple models is required to consolidate mechanistic evidence and enhance the robustness of experimental findings [6,23,27,28,29].

Significant gaps remain. Biomarkers of blood-CSF barrier dysfunction in humans are not yet sufficiently validated, and experimental protocols lack standardization, limiting comparability across studies [6,23,27,31,32]. The development of more complex models, including vascularized organoids, multicellular microfluidic systems, and immune-competent BCSFB models, represents a priority for advancing the field [23,27,28]. In addition, computational approaches addressing CSF flow dynamics, molecular transport, and compartment-specific inflammatory signaling may contribute to a more precise understanding of BCSFB function under physiological and pathological conditions [6,8,23,27].

### 3.9. Knowledge Gaps and Future Directions

Despite increasing interest in the BCSFB as a potential interface in viral neuroinvasion, major knowledge gaps continue to limit interpretation of its role in arboviral encephalitis. A central unresolved question is which arboviruses use the choroid plexus or BCSFB as a true route of CNS entry, and under which conditions this occurs. Current evidence supports direct involvement most clearly for ZIKV and, more recently, SFV [10,11]. For WNV, JEV, TBEV, and several other neurotropic arboviruses, the contribution of the choroid plexus remains suggestive or insufficiently tested [1,2,3,4,5]. A second major gap concerns viral tropism and receptor usage within the choroid plexus. Specific receptors or entry factors have been identified for some viral systems, such as VLDLR in SFV neuroinvasion through the BCSFB [11]. However, systematic validation across arboviruses is lacking [1,2,6,10,11]. In many cases, receptor expression in choroid plexus epithelial or stromal cells supports biological plausibility, but expression alone does not prove functional involvement in viral entry [6,23,25]. Future studies should combine receptor mapping, loss-of-function approaches, infection assays, and in vivo validation to define whether candidate receptors are required for BCSFB-mediated neuroinvasion [10,11,23,27]. The relationship between the BBB and BCSFB also remains unresolved. These barriers are anatomically and functionally distinct, but they may act in parallel or sequentially during infection [6,8,9,12]. CSF abnormalities, barrier leakage, and neuroinflammation may reflect primary BCSFB dysfunction, secondary BBB disruption, systemic inflammation, or combined effects across multiple CNS interfaces [1,2,6,12,31,32]. Current experimental and clinical tools are not sufficient to separate these possibilities with precision [6,12,31,32].

Another important limitation is the lack of validated biomarkers of BCSFB dysfunction in human arboviral encephalitis. Routine CSF findings, including viral RNA, intrathecal antibodies, pleocytosis, protein elevation, and inflammatory mediators, can support the diagnosis of neuroinvasive disease but do not define the route of CNS entry [31,32]. Future work should aim to identify barrier-specific biomarker panels that can distinguish BBB injury from BCSFB dysfunction and correlate these signatures with imaging, viral kinetics, and tissue-level pathology [6,12,13,24,31,32]. Model limitations also remain substantial. Two-dimensional in vitro systems provide mechanistic control but lack immune, vascular, and stromal complexity [23,26,27]. Organoids reproduce some aspects of choroid plexus epithelial organization but often lack mature vascularization and immune components [27,28]. Organ-on-chip systems can incorporate flow and multicellular interactions but remain technically demanding and incompletely standardized [23,27]. Animal models allow systemic analysis but are affected by species-specific differences in barrier biology and immune responses [1,6,29]. No single model is sufficient; integrated approaches will be required [6,23,27,28,29].

Future studies should prioritize longitudinal and cell-type-resolved approaches. These should include temporal tracking of viral load in blood, CSF, choroid plexus, meninges, and brain parenchyma; mapping of infected and activated cell populations; functional assays of barrier permeability; receptor validation; and integration of single-cell transcriptomics, spatial transcriptomics, proteomics, and CSF immune profiling [6,10,11,12,13,24,27]. Such approaches are needed to determine whether BCSFB involvement is primary, secondary, contributory, or incidental in each arboviral system [1,2,6,10,11].

## 4. Conclusions

BBB-centered models have been indispensable for understanding arboviral neuroinvasion, but they do not fully capture the range of anatomical interfaces that may participate in CNS entry and neuroinflammatory amplification [1,2,6]. The choroid plexus and the BCSFB deserve more explicit consideration within this framework, not as replacements for the BBB, but as additional interfaces whose relevance likely varies according to viral species, host context, receptor usage, immune status, and stage of infection [6,9,10,11,12,13]. At present, the evidence remains clearly uneven. Direct experimental support for BCSFB involvement is strongest for ZIKV and has recently been strengthened by studies of SFV [10,11]. In contrast, for other major neurotropic arboviruses, including WNV, JEV, and TBEV, the available evidence remains largely indirect or unresolved and does not yet support assigning the choroid plexus a universal or primary role in CNS entry [1,2,3,4,5].

The main value of the BCSFB framework is therefore not to replace one dominant paradigm with another, but to broaden the anatomical and mechanistic landscape through which arboviral neuroinvasion is interpreted [1,2,6,12]. A more accurate model should consider the BBB, BCSFB, meninges, peripheral nerves, immune-cell trafficking, and inflammatory signaling as potentially complementary contributors, while judging the role of each interface according to the strength of the available evidence [1,2,6,12,13]. Moving the field forward will require direct mechanistic studies that define viral tropism within the choroid plexus, clarify the temporal sequence of barrier crossing and CSF dissemination, identify receptor-dependent entry pathways, and determine whether BCSFB dysfunction is primary, secondary, contributory, or incidental during encephalitic disease [6,10,11,12,13,24,27]. Until such evidence is available across a broader range of viruses, the choroid plexus should be viewed as an underexplored and biologically plausible component of the neuroinvasion landscape, but not as a universally established gateway for arboviral CNS entry [1,2,6,10,11].

## Data Availability

No new data were created or analyzed in this study. Data sharing is not applicable to this article.

## Abbreviations

The following abbreviations are used in this manuscript:

**Abbreviation**	**Definition**
BBB	Blood-brain barrier
BCSFB	Blood-cerebrospinal fluid barrier
CCL2	C-C motif chemokine ligand 2
CNS	Central nervous system
CSF	Cerebrospinal fluid
CXCL10	C-X-C motif chemokine ligand 10
FcRn	Neonatal Fc receptor
IFN	Interferon
IFN-β	Interferon beta
IFN-γ	Interferon gamma
IgG	Immunoglobulin G
IgM	Immunoglobulin M
IL-1β	Interleukin 1 beta
IL-6	Interleukin 6
ISGs	Interferon-stimulated genes
JAK1	Janus kinase 1
JAK2	Janus kinase 2
JEV	Japanese encephalitis virus
LRP2	Low-density lipoprotein receptor-related protein 2
RNA	Ribonucleic acid
RT-PCR	Reverse transcription polymerase chain reaction
SFV	Semliki Forest virus
STAT1	Signal transducer and activator of transcription 1
STAT2	Signal transducer and activator of transcription 2
TBEV	Tick-borne encephalitis virus
TNF-α	Tumor necrosis factor alpha
TYK2	Tyrosine kinase 2
VLDLR	Very low-density lipoprotein receptor
WNV	West Nile virus
ZIKV	Zika virus
